# Vestibular Atelectasis: A Narrative Review and Our Experience

**DOI:** 10.3390/audiolres15030061

**Published:** 2025-05-18

**Authors:** Andrea Tozzi, Andrea Castellucci, Salvatore Martellucci, Pasquale Malara, Michael Eliezer, Giuseppe Ferrulli, Rosanna Rita Ruberto, Pasquale Brizzi, Enrico Armato, Alessio Marchetti, Daniele Marchioni, Angelo Ghidini, Claudio Moratti

**Affiliations:** 1 Department of Otorhinolaryngology-Head and Neck Surgery, University Hospital of Modena, 41125 Modena, Italy; andreatozzi29@gmail.com (A.T.); dottorgiuseppeferrulli@gmail.com (G.F.); daniele.marchioni@unimore.it (D.M.); 2 ENT Unit, Department of Surgery, AUSL-IRCCS di Reggio Emilia, 42123 Reggio Emilia, Italy; andrea.castellucci@ausl.re.it (A.C.); angelo.ghidini@ausl.re.it (A.G.); 3 ENT Unit, Santa Maria Goretti Hospital, Azienda USL di Latina, 04100 Latina, Italy; dott.martellucci@gmail.com; 4 Audiology & Vestibology Service, Centromedico, 6500 Bellinzona, Switzerland; pasmalara@gmail.com; 5 Neuroradiology Unit, Department of Diagnostic and Functional Neuroradiology and Brain Stimulation, 15-20 National Vision Hospital, Paris University Hospital Center, 75012 Paris, France; mcheliezer@gmail.com; 6 Audiology and Ear Surgery Unit, AUSL-IRCCS di Reggio Emilia, 42123 Reggio Emilia, Italy; rosannarita.ruberto@ausl.re.it (R.R.R.); pasquale.brizzi@ausl.re.it (P.B.); 7 Department of Neurosciences, University of Padova, 35100 Padova, Italy; 8 Vertigo Center, Poliambulatorio Chirurgico Modenese, 41125 Modena, Italy; alessio.marchetti@poliambulatoriopcm.it; 9 Department of Neuroradiology, AUSL-IRCCS di Reggio Emilia, 42123 Reggio Emilia, Italy; claudio.moratti@ausl.re.it

**Keywords:** vestibular atelectasis, Menière disease, MRI, Tullio phenomenon, Hennebert sign, intralabyrinthine schwannoma, endolymphatic hydrops

## Abstract

Vestibular atelectasis (VA) is a rare clinical entity characterized by a collapse of the endolymphatic space resulting in vestibular loss with the possible onset of positional and/or sound/pressure-induced vertigo. It could be idiopathic or secondary to other inner-ear diseases including Meniere’s disease (MD). A collapse of the membranous labyrinth involving the semicircular canals (SCs) and the utricle represents its distinctive histopathological feature. While specific radiological patterns consistent with VA have been described on contrast-enhanced MRI with delayed acquisitions, an impairment of the blood–labyrinthine barrier (BLB) could be detected in several disorders leading to vestibular loss. We conducted a narrative review of the literature on VA focusing on the putative pathomechanisms accounting for positional and sound/pressure-induced nystagmus despite unilateral vestibular loss (UVL) in this condition, providing two novel cases of VA. Both patients presented with a clinical picture consistent with unilateral MD that rapidly turned into progressive UVL and positional and/or sound/pressure-induced vertigo. In both cases, the posterior SC was initially impaired at the video-head impulse test (vHIT) and both cervical and ocular VEMPs were initially reduced. Progressively, they developed unsteadiness with paretic spontaneous nystagmus, an impairment also for the lateral and anterior SCs, caloric hypo/areflexia and VEMPs areflexia. They both exhibited ipsilesional nystagmus to sound/pressure stimuli and in one case a persistent geotropic direction-changing positional nystagmus consistent with a “light cupula” mechanism involving the lateral SC of the affected side. A collapse of the membranous labyrinthine walls resulting in contact between the vestibular sensors and the stapes footplate could explain the onset of nystagmus to loud sounds and/or pressure changes despite no responses to high- and low-frequency inputs as detected by caloric irrigations, vHIT and VEMPs. On the other hand, the onset of positional nystagmus despite UVL could be explained with the theory of the “floating labyrinth”. Both patients received contrast-enhanced brain MRI with delayed acquisition exhibiting increased contrast uptake in the pars superior of the labyrinth, suggesting an impairment of the BLB likely resulting in secondary VA. A small intralabyrinthine schwannoma was detected in one case. VA should always be considered in case of positional and/or sound/pressure-induced vertigo despite UVL.

## 1. Introduction

Vestibular Atelectasis (VA) is a rare entity firstly described in 1988 by Merchant and Schuknecht in histopathological studies on the temporal bones [1]. It is referred to as the collapse of the membranous labyrinth, in particular, the ampullae of the semicircular canals (SCs) and the utricle (i.e., superior labyrinth), resulting in an array of vestibular symptoms and signs. The authors distinguished two different clinical entities of VA: a primary type with an insidious or paroxysmal onset and a secondary type, considered as the histopathological result of other vestibular disorders. According to the classification, the clinical presentation could vary between a rapid onset of vestibular symptoms, similar to a vestibular neuritis (VN) with neither cochlear nor neurological symptoms, followed by chronic disequilibrium, and a more insidious onset leading to chronic unsteadiness.

Wenzel et al. first proposed the association between a complete bilateral vestibular areflexia associated with sound- and/or pressure-induced vertigo/unsteadiness (Tullio phenomenon and Hennebert sign, respectively), considering the contact between the collapsed membranous labyrinth and the stapedial footplate as a possible explanation of the phenomenon [2]. More recently, several cases of bilateral VA characterized by the Tullio phenomenon and normal cervical vestibular-evoked myogenic potential (cVEMPs) have been described in the literature [2,3,4,5]. Furthermore, a clinical investigation on a recent series of unilateral VA has been reported [6]. However, most of these investigations only advanced clinical hypotheses, while VA was not demonstrated in vivo. Eliezer et al. were the first to document the collapse of the membranous labyrinth in vivo using delayed post-contrast three-dimensional fluid attenuated inversion recovery (3D-FLAIR) 3T magnetic resonance imaging (MRI) sequences in patient presenting with unilateral vestibular loss (UVL) or bilateral vestibulopathy (BVP) [7,8]. They considered the endolymphatic space as collapsed when the utricle and at least two SC ampullae could not be visualized or were barely detectable on MRI (e.g., total collapse and partial collapse, respectively). Notably, it has been demonstrated that the volumetric variation in the endolymphatic spaces can also be visualized using lower-resolution devices, such as 1.5 T MRI [9,10].

Despite the limited number of records on VA, only the first paper by Merchant and Schuknecht specifically addressed the secondary forms. In fact, they reported VA secondary to aging, temporal bone fractures, surgical procedures (e.g., stapedectomy and mastoidectomy), and endolymphatic hydrops (EH).

Meniere’s disease (MD) is a clinical condition characterized by the combination of episodic vertigo, fluctuating sensorineural hearing loss (SNHL) and tinnitus or aural fullness [11]. The anatomical condition accounting for MD is thought to be EH. MD can exhibit various clinical staging. The first stage of the disease is characterized by fluctuating low-frequency SNHL with vertigo spells, generally not associated with labyrinthine hypofunction. At the beginning, the Lermoyez variant may occur, where the onset of vestibular symptoms leads to auditory improvement [12,13]. The following stages of MD are characterized by a progressive worsening of hearing function and the onset of partial or total labyrinthine impairment, with the possible occurrence of Tumarkin otolithic crises [14]. Finally, a severe flat SNHL associated with a global UVL and unsteadiness distinguish the last stage of the disease.

Intralabyrinthine schwannoma (ILS) is a rare benign tumor arising from the Schwann cells surrounding the terminal branches of the cochlear or vestibular nerves within the membranous labyrinth [15]. It can clinically present with progressive, fluctuating or acute SNHL, tinnitus, dizziness and vertigo, sometimes accompanied by MD-like attacks. The array of symptoms mimicking MD may contribute to a delayed diagnosis of this lesion [16].

To our knowledge, no studies in the literature have investigated the progression towards VA from an instrumental point of view. In this paper, we conducted a narrative review of the literature on VA and provided two additional cases presenting with a clinical picture consistent with unilateral MD (in one case an ILS mimicked MD) and then developing sound/pressure-induced vertigo despite a progressive UVL likely due to VA. We also aimed to provide possible pathophysiological explanations for the symptoms and signs of this clinical condition.

## 2. Materials and Methods

Search Sources and Strategy

An online search on VA was conducted across PubMed and Embase, including both English and non-English language articles. The keyword used was “Vestibular Atelectasis”. Additionally, a manual search of all the references from the selected articles was conducted. All studies on VA presenting case reports, case series and original articles were included. Similarly, all clinical/radiological/histopathological studies and all studies on animal models were included. The search was updated to 31 December 2024. Being a narrative review of the literature, neither strict inclusion criteria were adopted, nor ethical approval was required.

Case 1 Presentation

**Figure 1:**
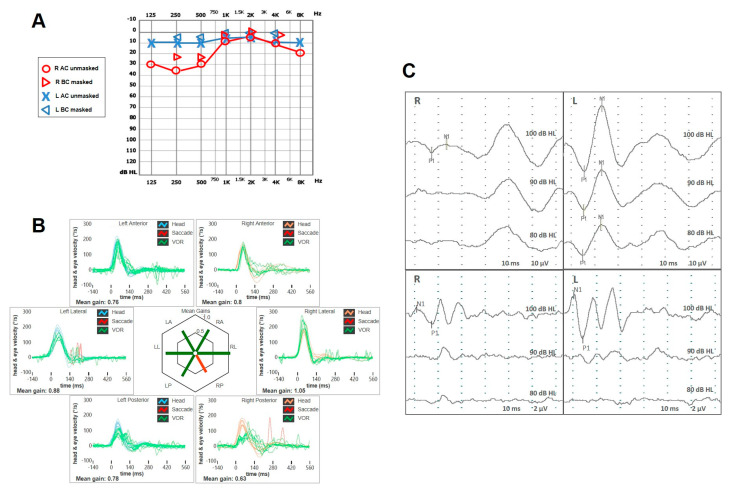
Early instrumental assessment of patient n. 1. (**A**) Pure-tone audiometry with slight low-frequency SNHL on the right side. (**B**) vHIT with selective slight VOR-gain impairment for the right PSC. Blue lines represent head impulses exciting left canals, orange lines correspond to impulses for right canals, green lines represent eye movements induced by the activation of the VOR following each impulse and red lines correspond to corrective saccades. Mean value of VOR gain (eye velocity/head velocity) is reported for each canal. The hexagonal plot in the center of the figure summarizes mean VOR gains for each canal; normal gains are shown in green and deficient gains are shown in red. Gains are considered normal if more than 0.8 for lateral SCs and more than 0.7 for vertical SCs. (**C**) cVEMPs (**above**) and oVEMPs (**below**) with a significant reduction in amplitudes on the right side (AR > 35%).

**Figure 2:**
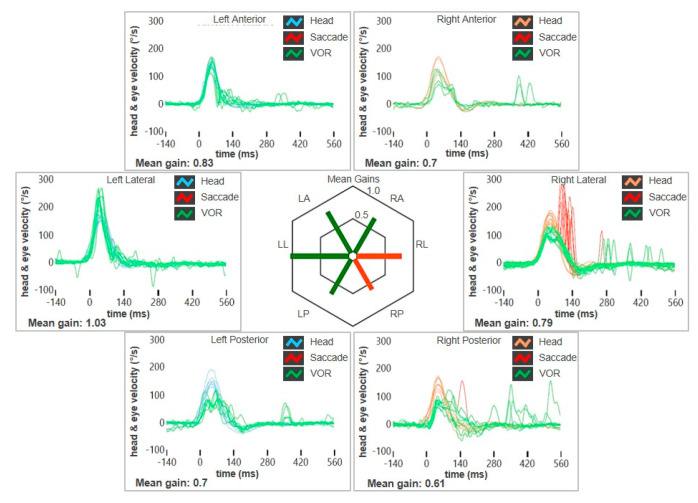
vHIT data of patient n. 1 at the relapse of dizziness. Reduced VOR gain values for the right LSC and PSC with corrective saccades can be detected.

**Figure 3:**
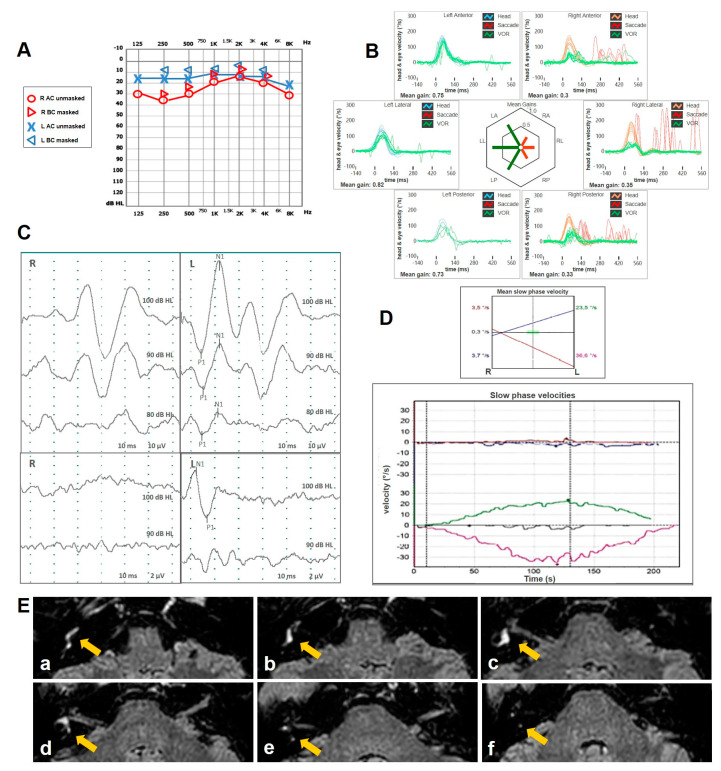
Final instrumental follow up and radiological data of in patient n. 1. (**A**) Pure-tone audiometry with slight low-frequency SNHL on the right side. (**B**) vHIT with severe reduction in the VOR gain for all the right SCs. (**C**) cVEMPs (**above**) and oVEMPs (**below**) showing no detectable responses on the right side. (**D**) BCT with caloric areflexia on the right side. (**E**) FLAIR images from 1.5 T brain MRI with delayed acquisitions: sequences from the lower to the upper part of the inner-ear (a–f) highlighting on the right side an intralabyrinthine enhancement (yellow arrow) of the vestibule, utricle, semicircular canals and basal turn of the cochlea.

A 49-year-old female with a long-lasting history of right-sided MD was addressed to our institution for recurrence of vertigo after 8 years of quiescence. She reported arterial hypertension, while neither migraine nor history of head trauma/barotrauma was reported. At the onset of MD, she initially experienced right fluctuating low tone-SNHL and tinnitus, sporadic vertigo spells and slight dizziness. The video head impulse test (vHIT) showed a slight posterior SC (PSC) hypofunction on the right side and both air-conducted (AC) cVEMP and ocular VEMP (oVEMPs) amplitudes showed a slight decrease on the right side (Figure 1). A standard Gadolinium-enhanced brain MRI was normal. After 8 years, she was eventually evaluated for a relapse of dizziness. No hearing fluctuations were reported. A mild spontaneous left-beating nystagmus enhanced by the skull vibration-induced nystagmus test (SVINT) could be detected with the video-Frenzel goggles (Video S1). The vHIT revealed a reduced gain of the vestibulo-ocular reflex (VOR) for the lateral SC (LSC) and the PSC of the right side (Figure 2). The patient immediately began steroid therapy (1 week intravenous 1 mg/kg dexamethasone followed by oral tapering for 1 additional week) and oral diuretics (acetazolamide 250 mg/day). One week later, she was re-evaluated due to a deterioration of unsteadiness and to the onset sound-induced vertigo. No more spontaneous nystagmus could be detected, while left-beating nystagmus could be elicited by head shakings and SVINT, and a tiny right-beating nystagmus could be elicited by loud sounds on the right side. While hearing function was basically stable, a global worsening of the VOR gain for all the SCs on the right side was observed on vHIT, including the anterior SC (ASC). AC cVEMPs and oVEMPs were reassessed, confirming a complete loss of the potentials on the right side and a bithermal caloric test (BCT) confirmed a right-sided areflexia (Figure 3A-D). A high-resolution CT (HRCT) scan of the temporal bones was unremarkable, while a Gadolinium-enhanced brain MRI (1.5 T) with 4 hour-delayed FLAIR inner-ear acquisitions revealed an increased contrast enhancement at the superior labyrinth and at the cochlear basal turn of the right side, suggesting a blood–labyrinthine barrier (BLB) impairment (Figure 3E). The patient began oral treatment with 48 mg/day Betahistine and was sent to vestibular rehabilitation.

Case 2 Presentation

**Figure 4:**
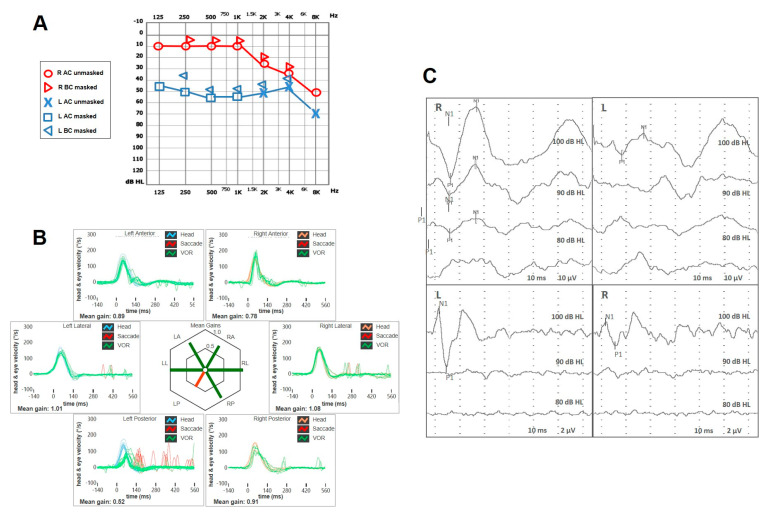
Early instrumental assessment of patient n. 2. (**A**) Pure tone audiometry with slight high-frequency SNHL on the right side and mild–severe flat SNHL on the left side. (**B**) vHIT with selective slight VOR gain impairment for the left PSC. (**C**) cVEMPs (above) and oVEMPs (below) with significant reduction in amplitude on the left side (AR > 35%).

**Figure 5:**
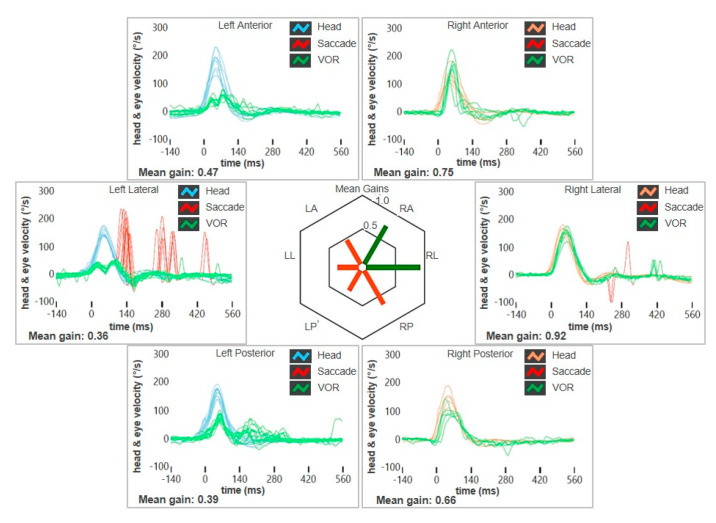
vHIT data of patient n. 2 at the onset of both positional and sound/pressure-induced vertigo. Reduced VOR gain values for the left SCs (in particular for the PSC and the LSC) with a slight hypofunction also for the contralateral PSC can be detected.

**Figure 6:**
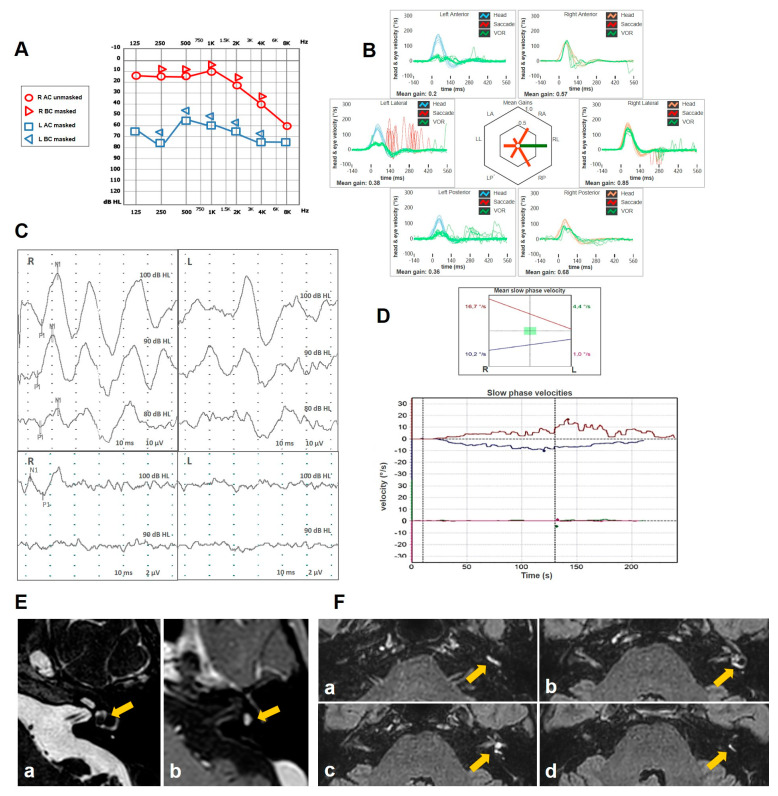
Final instrumental follow up and radiological data of in patient n. 2. (**A**) Pure-tone audiometry with a slight impairment of the flat SNHL on the left side. (**B**) vHIT with severe reduction in the VOR gain for all the left SCs and a slight impairment for the contralesional vertical SC. (**C**) cVEMPs (above) and oVEMPs (below) showing no detectable responses on the left side. (**D**) BCT with caloric areflexia on the left side. (**E**) Brain MRI: T2 (**a**) and T1 with Gadolinium (**b**) highlighting a small ILS on the left side (yellow arrow). (**F**) FLAIR images from 1.5 T brain MRI with delayed acquisitions: sequences from the lower to the upper part of the inner-ear (**a**–**d**) highlighting intralabyrinthine enhancement (yellow arrow) involving all portions of the perilymphatic space of the left side.

A 58-year old male with a clinical picture consistent with left-sided MD presented to our attention for recurrent vertigo spells despite a long-lasting treatment with Betahistine and diuretics. His clinical history was unremarkable except for congenital anisocoria. His standard brain MRI was normal. His pure-tone audiometry showed a slight high-frequency SNHL on the right side and a moderate-to-severe left SNHL on the left side. A comprehensive vestibular evaluation revealed a slight hypofunction for the left PSC at the vHIT and an ipsilateral reduction for both AC cVEMPs and oVEMPs (Figure 4). Even though 5 intratympanic dexamethasone injections were administered weekly in his left ear, his vestibular symptoms progressively worsened, including severe unsteadiness and the onset of both positional and sound/pressure-induced vertigo. No changes in auditory symptoms were reported. A spontaneous right-beating nystagmus enhanced by the SVINT was detected on video-Frenzel examination. Nasal and glottic Valsalva maneuvers induced a clear left-beating nystagmus. Similarly, the patient exhibited a slight left-beating nystagmus in response to loud sounds on the left side. A persistent geotropic direction-changing positional nystagmus was evoked at the supine head roll test (SHRT). Apparently, nystagmus amplitude was greater on the right side (Video S2). A significant reduction in all the left SCs, particularly for the PSC and the LSC, was found at the vHIT (Figure 5). The patient immediately started intravenous steroid therapy. Two weeks later, he was re-evaluated. Spontaneous nystagmus subsided and the hearing function on the left side was slightly worsened. The vHIT demonstrated a further decrease in the VOR gain values for all the SCs on the left side and both cVEMPs and oVEMPs left were absent on the left side. Additionally, BCT showed left vestibular areflexia. While temporal bone HRCT was unremarkable, a Gadolinium-enhanced brain MRI (1.5 T) revealed a 3 mm ILS. Four-hour delayed FLAIR sequences highlighted an intralabyrinthine enhancement (yellow arrow) involving all portions of the perilymphatic space of the left side (Figure 6). The patient was immediately sent to vestibular rehabilitation.

Written informed consent for the publication of all data, images and videos was obtained from both patients.

## 3. Discussion

VA is still considered a rare entity with few scientific records reported in the literature. 

The histopathological description of VA by Merchant and Schuknecht in 1988 represents the first study on this novel clinical entity. The elegance of their work lay in the ability to identify histopathological criteria among 426 temporal bones to distinguish true VA from an artifact resulting from specimen preparation. In five out of eight cases of VA (62.5%), a history consistent with unexplained vertigo was found. Consequently, they defined this novel histopathological entity—likely responsible for the clinical presentation of affected patients—as primary VA. All cases had unilateral VA and were characterized by a collapse of the superior labyrinth (i.e., SCs and utricle). The same authors were also the first to describe six cases of secondary VA due to an underlying vestibular pathology (VN, EH, temporal bone fracture or iatrogenic causes). In these patients, VA symptoms might have been masked by the pre-existing pathology or remained undetected due to collapse occurring in an already significantly altered labyrinth. The authors suggested a dual relationship with EH: the primary collapse of the superior labyrinth could obstruct the longitudinal endolymphatic flows, or alternatively, the pressure from EH itself could act as the “primus movens” for the membrane rupture [1]. The possible connection with EH was also hypothesized by Nadol and Schuknecht, who described a collapse of the superior labyrinth while investigating the potential causes of vertigo in the elderly. This phenomenon could be explained as a rupture of the saccule in the presence of an incomplete utricular-saccular valve, leading to an endolymphatic outflow [17]. In the series of temporal bones analyzed by Khetarpal, who aimed to identify a morphological correlate in patients with idiopathic sudden SNHL presenting with or without vertigo, the Author described two cases (one per group) of saccular atelectasis, without identifying any significant correlation with his study data [18]. Nadol included VA in the differential diagnoses of VN, as it could also lead to acute vertigo attacks with autonomic symptoms and sudden SNHL. In both conditions, the recovery could need a long period of time. However, he noted that the exacerbation of unsteadiness associated with head movements was rarely reported in cases of VA [19]. In 2005, Viana et al. first described a case of bilateral idiopathic Dandy syndrome. This syndrome is characterized by BVP, oscillopsia, and SNHL in some cases, and it exhibited bilateral VA involving the three SCs associated with a global depletion of the labyrinthine receptors. This histopathological profile perfectly aligned with the criteria defined by Merchant and Schuknecht [20]. Histopathological studies have led to the hypothesis of a pathophysiological mechanism for VA that is closely linked to EH [1,17]. It is well known that a partial or total collapse of the membranous labyrinth could occur in the late stages of MD. However, some authors suggested that this collapse could account for the improvement of acute vertigo spells that typically distinguish the late stages of the disease, contrary to the findings reported by Merchant and Schuknecht [21].

Further insights into the pathogenic mechanisms of VA have been provided through studies on animal models. Nomura et al. observed both SCs and otolithic collapse in histological specimens through experimentally inducing a perilymphatic fistula (PLF) in guinea pigs [22]. The same animals underwent BCT, which revealed either normal responses, areflexia or irregular responses, consistent with findings in patients affected by PLF [22,23,24]. In a previous study conducted by the same group, the authors had already observed a variable degree of collapse of the superior labyrinth in 50% of specimens with a PLF, while 25% exhibited cochlear hydrops [25]. They also found both paretic nystagmus and/or positional nystagmus (in particular when the affected ear was in the undermost position) in both animal models and human subjects with PLF resulting in a collapse of the membranous labyrinth [22,23,24,26]. Moreover, they defined two possible scenarios: in the case of a total collapse, the sensory organs were decoupled from the surrounding structures, whereas in the case of partial collapse, the displacement of the partly collapsed labyrinth could stimulate the sensory organs. The authors concluded that the variability in caloric responses, from normal pattern and duration to canal paresis, could therefore be explained by the degree of labyrinthine collapse. While the first condition was reconducted to VA, the latter condition was defined as “floating labyrinth”, which was supposed to represent an early stage of VA that could either progress to VA or return to its normal condition [22,23,24]. According to this hypothesis, when a collapse occurs, given that the membranous labyrinth in the pars superior is supported by the trabecular mesh, changes in pressure within the perilymph and/or cerebrospinal fluid (CSF) may lead to movement of the membranous labyrinth. Specifically, if the collapsed wall comes into contact with the otolithic membrane or the sensory epithelium of the ampullary crest, its drifting may stimulate sensory cells in the utricle or the SCs ampullae accounting for unsteadiness [22,23,24]. On the other hand, a floating labyrinth might also account for the positional nystagmus detected in these subjects. In fact, positional nystagmus may occur due to a disruption in the interaction between the otolithic organs and the SCs, caused by changes in the movement of the inner-ear fluids. The partially collapsed membranous labyrinth becomes attached to the otolith of the utricular macula and the ampullary crests. Perilymph flow may cause the collapsed membranous labyrinth to shift, thereby stimulating the sensory cells [22,23,24].

Another distinctive animal model was proposed by Kawaguchi et al. using the “waltzing guinea pig”, a model characterized by the early degeneration of the vestibular apparatus, likely related to alterations in ion transport mechanisms involved in endolymph homeostasis. In these specimens, a degeneration of the dark cell region resulted in a labyrinthine collapse [27].

In the present study, we reported two cases of secondary VA and, to the best of our knowledge, it represents the only report documenting the evolution of the vestibular function towards a secondary VA in patients with MD and ILS. While both patients developed a rapidly progressively UVL on the affected side with paretic spontaneous nystagmus enhanced by the SVINT, similarly to an acute stage of VN [28,29], patient n. 2 also presented with a persistent geotropic direction-changing positional nystagmus at the SHRT consistent with a “light cupula” mechanism of the affected LSC. In fact, even though positional nystagmus was apparently stronger on the healthy side, the underlying baseline right-beating spontaneous nystagmus likely mitigated the positional geotropic nystagmus on the left side, which should be expected to be stronger than the opposite side in accordance with Ewald’s laws. In fact, in light cupula, due to the anatomical position of the LSC and the density ratio between the cupula and the surrounding endolymph, the cupula of the affected LSC bends towards the ampulla, resulting in a persistent excitation of the LSC afferents when the head is rotated towards the affected side on the supine position, while it bends away from the ampulla, inhibiting the LSC afferents, in the opposite head position [30,31]. Moreover, in case of UVL involving the utricular macula, spontaneous paretic nystagmus increases its amplitude when the affected ear is in the undermost position, likely due to an asymmetrical activity of the tonic afferents coming from both utricles [32]. Therefore, the enhanced right-beating spontaneous nystagmus in left positioning could have further reduced the positional nystagmus in the same position. The “light cupula” theory has been described in cases with damage to the BLB, as it might result in the leakage of plasma proteins into the inner-ear fluid, increasing the buoyancy of the endolymph [33,34]. Similar persistent geotropic direction-changing positional nystagmus has been detected in other inner-ear pathologies such as labyrinthine fistula and acute otitis media where the penetration of toxic agents and/or inflammatory mediators into the affected SC and subtle mass-induced pressure changes on the membranous labyrinth have been assumed as putative mechanisms [35,36,37]. Another possible scenario accounting for persistent geotropic direction-changing positional nystagmus that might fit the present case is the onset of a difference in the density between perilymph and endolymph. In fact, it has been assumed that if perilymph becomes denser than endolymph, the SCs filled with endolymph develop an increased buoyancy under gravity. This buoyancy, combined with the deformable nature of the membranous labyrinth, can cause the membranous ducts to deform, displacing the endolymph and deflecting the cupula, leading to a persistent geotropic direction-changing positional nystagmus [38]. This latter explanation likely matches the “floating labyrinth” theory, which has been assumed as the pathomechanism accounting for positional nystagmus with the affected ear undermost in VA [22,23,24].

Another common finding in both patients herein described is sound- and/or pressure-induced nystagmus. This sign, originally linked to congenital syphilis and labyrinthine fistulas, has since been observed in various conditions. Even though it has become a distinctive symptom for third window syndromes like ASC dehiscence [39,40], over the past 60 years, studies have shown the onset of sound- and/or pressure-induce vertigo in a number of clinical conditions including congenital deafness, PLF and MD [41,42]. Nadol linked the phenomenon to fibrous adhesions in the inner ear, which has been observed in conditions such as viral labyrinthitis and MD [41]. These findings suggest a potential link between the Tullio phenomenon and/or Hennebert signs and EH, where the distension of the labyrinth makes contact with the stapes footplate. The Tullio phenomenon with BVP exhibiting a pure horizontal nystagmus in response to loud sounds and in the absence of SC dehiscence was previously described in a single patient by Acierno et al. [43], and more recently by Kaski et al. [40] in three patients who underwent a thorough audio-vestibular testing. These phenomena, which were not reported in the original study by Merchant and Schuknecht, were firstly linked to VA by Wenzel et al. [2]. The authors proposed an explanation based on labyrinthine collapse and subsequent vestibular hypofunction. Specifically, the collapsed utricle would move closer to the stapedial footplate, making it mechanically excitable by sound or intense auditory stimuli. In both cases herein reported, increased intratympanic pressure resulting from nasal Valsalva and loud sounds and increased CSF pressure resulting from glottic Valsalva might have led the collapsed labyrinth to drift within the surrounding perilymph, resulting in the activation of the SC afferents of the affected ear. The vestibular assessment of the patients reported by Wenzel et al. revealed a certain degree of dissociation between high-frequency responses and caloric responses: while BCT showed complete areflexia, high-frequency responses (vHIT) were only reduced by 20–30% [2]. This discrepancy led the authors to suggest that the collapse of the membranous labyrinth acted as a plug, allowing for a partial response to high-frequency stimuli while completely blocking low-frequency responses. Nevertheless, according to the studies on inner-ear physiology, nystagmus is generated by cupular displacements which are primarily encoded by type II hair cells and regular canal afferents [44,45,46]. This apparently contradictive finding may be explained by a possible “pseudo-areflexia” of the ampullary hair cells that encodes angular accelerations due to a partial collapse of the membranous labyrinth, at least in the cases that we report. Specifically, the presence of detectable sound/pressure-induced nystagmus, and similarly positional nystagmus, despite SC impairment on vHIT and BCT, indicates that at least type II hair cells and regular canal afferents are functionally spared and can be activated by strong stimuli (like loud sounds and pressure changes), while they cannot be stimulated by endolymphatic convective flows nor by fluid expansion resulting from caloric irrigations. On the other hand, the presence of a paretic spontaneous nystagmus together with a complete SC impairment on vHIT and VEMPs in both patients implies that a certain degree of labyrinthine loss has occurred, so that one might hypothesize a functional dissociation between hair cells encoding high- (damaged) and low-frequency (spared) inputs [37].

Another key difference between Wenzel’s study and that of Merchant was the bilaterality of the VA. This case series raised concerns within the scientific community, particularly regarding the true prevalence of VA, its etiology, and its possible explanation through other well-known conditions, such as SC dehiscence or near-dehiscence syndrome [47]. The main limitation of the proposed studies was the retrospective nature of data collection, which was based on histopathological examination. Similarly, case reports published in more recent years focused on the clinical and vestibular presentation, but the definitive confirmation of VA remained impossible without autoptic examination [3,4,5,48].

A key contribution to the definition of VA has been provided by Eliezer and the French school [7]. In a retrospective study published in 2019, they first described VA in vivo in four cases: they used new imaging acquisitions in MRI (in particular, delayed post-contrast 3D-FLAIR sequences) which are currently used for the study of EH [49,50,51,52]. Based on histopathological definitions, the authors classified VA as total or partial, depending on the absent or partial visibility of at least two SC ampullae and the utricle. Additionally, the dissociation of VOR responses at high and low frequencies was consistent with the previously described findings in VA. The study mainly focused on the primary forms of VA, excluding patients with pre-existing vestibulopathies (EH, PLF or BLB dysfunction). However, the authors acknowledged that in acute stages, certain vestibulopathies—particularly VN—can present with MRI alterations of the BLB which might evolve towards a UVL [53]. The cases described by these authors were exclusively unilateral, thus failing to confirm Wenzel’s hypothesis of VA as a marker or cause of idiopathic BVP [7]. This study was followed by the first case report identifying a suspected bilateral VA in vivo through MRI, and a series of patients diagnosed with BVP in which the diagnostic criteria for VA were met in 50% of patients [8,53]. Several pathophysiological mechanisms have been proposed by various authors to explain VA. One hypothesis suggests that a mutation in the KCNQ1/KCNE1 receptor, which is expressed by dark cells, could lead to a vestibular collapse. Since the saccule does not contain dark cells, it would remain unaffected by this process. Additionally, the administration of aminoglycosides or a long-standing vestibular disorder could result in secondary VA due to vestibular deafferentation. However, the authors argue that VA itself is primarily responsible for the clinical picture, as observed in a patient who underwent MRI just 10 days after symptom onset. Similarly, in subsequent studies focusing on unilateral VA cases, additional mechanisms were proposed, including a defective closure of Bast’s valve (which should lead to a decreased pressure in the endolymphatic compartment and subsequent atelectasis), alterations in membrane ion transporters of dark cells and dysfunctions in the vasopressin-aquaporin system: they all represent potential contributors to VA that have yet to be investigated [6]. Another interesting finding reported by Marc et al. in their study on unilateral VA is the presence of auditory symptoms in 40% of patients, particularly in acute cases. Recently, further investigations into autoptic studies of patients with VN and the temporal bone collection have renewed interest in VA as a finding, which was identified in post-mortem specimens [54].

Both cases herein reported patients presenting with a rapid onset of unsteadiness, paretic spontaneous nystagmus, impaired vHIT VOR gains on the lesioned side and MRI signs of impairment of the BLB. This clinical/radiologic scenario, which could be theoretically consistent with VN/labyrinthitis, is likely the first stage of a secondary VA. In fact, both patients had a history consistent with previous ipsilateral inner-ear lesion (MD/ILS) and developed a specific pattern of progression for SC impairment. In particular, at the beginning they both exhibited a selective slight lesion of the ipsilateral PSC at the vHIT and reduced AC VEMPs on the same side, which is considered consistent with MD [55,56,57,58]. Suddenly, both patients started to develop severe unsteadiness, paretic spontaneous nystagmus and a rapidly progressive onset of UVL with an ascending lesion pattern (involving the LSC first and finally the ASC at the vHIT) including an areflexia to BCT and both cVEMPs and oVEMPs. This kind of lesion pattern is similar to what has been detected in most VA cases [6,7,53] and in a recent case of possible PLF with an MRI detection of collapsed endolymphatic space [59]. In fact, in these conditions, the PSC represents the most affected SC while the ASC is the most frequently spared structure, opposite to VN where ASC and LSC represent the most commonly affected SCs [60,61,62]. The VOR-gain reduction for the vertical SCs on the healthy side that could be detected at the vHIT in the second case could be likely explained either by a loss of the peripheral push–pull mechanism coming from the functionally coupled contralateral vertical SCs which were severely impaired or with a central compensation mechanism due to cerebellar clamping [63,64]. Moreover, both patients developed sound/pressure-induced vertigo despite UVL, consistent with VA, which represent a sign that has never been described in VN. Similar “ascending” lesion patterns with preferred involvement of the PSC first and of the other SCs subsequently at the vHIT have been detected in age-related vestibulopathy [65,66,67], suggesting a possible link between presbyvestibulopathy and VA [17]. In both cases reported herein, the most likely pathomechanism for the rapidly progressive vestibular impairment and the relative clinical/instrumental scenario could be a VA secondary to EH that finally resulted in a rupture of the endolymphatic membranes. In the second patient, the ILS could have likely mimicked MD or might have caused EH. Unfortunately, a prolonged follow up over the months/years, which could potentially provide further data on the modification of the instrumental and radiological picture, reinforcing the diagnosis of VA, is lacking. Moreover, in both cases a 1.5 T MRI was adopted, with a possible reduction in the anatomical details compared to a 3 T MRI.

Since most studies on VA in the literature are either autoptic or radiological investigations, and only a few of them are focused on symptoms and findings and on the possible misdiagnosis, there is little quantitative analysis of the instrumental data in this review. Therefore, the information that could be extracted from the clinical studies is not enough to draw diagnostic algorithms, flowcharts or guidelines for VA based on the data from the literature, also considering the significant variability in symptoms and signs. This information would have undoubtedly contributed to a more comprehensive narrative and the analysis of these details would have certainly helped clinicians in gaining a better understanding of the differential diagnosis between VA and other inner-ear conditions such as VN and MD. We suggest that this aspect might represent a potential area for further investigation in future studies, in the hope that more research will focus on the diagnostic challenges associated with this rare condition.

Finally, the definition of VA as a new clinical entity has opened the door to the exploration of therapeutic strategies. Wenzel proposed the possibility of performing vestibular rehabilitation cycles or, in the most disabling cases, intratympanic gentamicin injections [2]. Similarly to acute UVL, intratympanic corticosteroids could also be considered [68]. Moreover, MRI may provide valuable information for identifying patients who could benefit from a vestibular implant, as the lesion primarily affects labyrinthine sensory receptors rather than the vestibular nerve, which could therefore respond to direct stimulation [69].

All the main studies on vestibular atelectasis are summarized in Table 1 in a chronological order.

## 4. Conclusions

In conclusion, VA represents a clinical condition that should be always considered when encountering a UVL that does not follow the usual progression or in case of fluctuating vestibulopathy that develops an insidious or a rapidly progressive functional impairment. Moreover, clinicians should be aware of the possibility of an underlying VA in case of positional and/or sound/pressure-induced vertigo despite vestibular loss. This narrative review contributes to the improved clinical understanding of a condition that has become a significant differential diagnosis.

## Figures and Tables

**Table 1:** Literature review on VA.

Authors	Year	Type of Article	Field of the Study	N° of Cases with VA	Site of VA	Brief Description	Vestibular Assessment
Merchant and Schuknecht [1]	1988	Original Article	Histopathological	11/426	Unilateral degeneration of the superior labyrinth (SCs + utricle)	First histopathological evidence of a new clinical entity. Distinction between primary and secondary variants and possible pathophysiological explanations.	BCT: different responses (either normal or irregular responses)
Nadol and Schuknecht [17]	1990	Original Article	Histopathological	1/14	Unilateral degeneration of the superior labyrinth (SCs + utricle)	Classification of the causes of vertigo in the elderly illustrated through clinical and histopathological cases.	/
Kehetarpal [18]	1991	Original Article	Histopathological	2	Unilateral degeneration of the inferior labyrinth (cochlea + saccule)	First description of cochleo-saccular atelectasis in patient with idiopathic sudden SNHL.	/
Nomura et al. [22]	1992	Original Article	Animal model	/	/	An experimentally induced PLF in guinea pigs causes a collapse of the membranous labyrinth.	Paretic spontaneous nystagmus and positional nystagmus with the lesioned ear undermost. BCT: different responses (either areflexia, normal responses or irregular responses).
Nomura et al. [26]	1992	Case Report	Clinical	/	/	Two patients with PLF.	Positional nystagmus with the lesioned ear undermost BCT: different responses (either normal or irregular responses).
Young et al. [24]	1992	Original Article	Animal model	/	Partial collapse (floating labyrinth) or total collapse (VA) of the membranous labyrinth	The term ‘floating labyrinth’ corresponds to partial VA.	Paretic spontaneous nystagmus and positional nystagmus with the lesioned ear undermost.
Young et al. [23]	1992	Original Article	Animal model	6/46	Partial collapse of the membranous labyrinth (floating labyrinth)	Floating labyrinth as an intermediate stage VA and a normal membranous labyrinth.	BCT: different responses (either areflexia, normal responses or irregular responses).
Nadol [19]	1995	Original Article	Histopathological	2	/	VA can be included in the differential diagnosis with VN.	/
Kawaguchi et al. [27]	2010	Original Article	Animal model	/	/	“Waltzing” guinea pig: a new model for studying VA. Labyrinthine collapse resulting in severe degeneration in the dark cell area.	/
Viana et al. [20]	2013	Original Article	Histopathological	1	Bilateral degeneration of all the SCs with saccule and utricle preservation	Idiopathic Dandy Syndrome. Reduction in neurons in Scarpa’s and Spiral’s ganglions.	/
Wenzel et al. [2]	2014	Original Article	Clinical	4	Bilateral SCs and utricular collapse associated with sound/pressure-induced vertigo and normal hearing	First description of sound/pressure-induced vertigo in VA. Pathophysiological explanation: dissociation between low- and high-frequency responses.	BCT: bilateral areflexia vHIT: bilateral VOR reduction for all the SCs
Finn et al. [3]	2018	Case Report	Clinical	1	BVP.	Sound/pressure-induced nystagmus, no hearing loss. BCT: bilateral areflexia. vHIT: bilateral VOR reduction for all the SCs cVEMPs: normal, oVEMPs: bilateral reduction.	
Heidenreich et al. [48]	2018	Original Article	Clinical	3	BVP with sound/pressure-induced vertigo	EcochG application in patients with BVP.	Sound/pressure-induced nystagmu. BCT: bilateral hypofunction. Rotatory chair: decreased gain vHIT: bilateral VOR reduction for all the SCs cVEMPs: normal.
Maslovara et al. [5]	2018	Case Report	Clinical	1	Bilateral VA	Sound/pressure-induced nystagmus. BCT: bilateral areflexia. cVEMPs: normal, oVEMPs: bilateral reduction.	
Roy et al. [4]	2019	Case Report	Clinical	1	Bilateral VA	Sound-induced nystagmus.	
Eliezer et al. [7]	2019	Original Article	Radiological	4/200	Unilateral VA with collapse of the superior labyrinth (SCs + utricle)	First description in vivo of VA with 3T -MRI and radiological definition of partial and total labyrinthine collapse.	BCT: unilateral areflexia. vHIT: VOR gain. reduction for LSC and PSC. cVEMPs: normal. oVEMPs: absent.
Eliezer et al. [8]	2019	Case Report	Clinical	1	Bilateral VA with collapse of the superior labyrinth (SCs + utricle)	First description in vivo of bilateral labyrinthine collapse. VA as a cause of BVP.	BCT: bilateral areflexia. vHIT: bilateral VOR reduction for all the SCs. oVEMPs: bilaterally absent.
Eliezer et al. [53]	2020	Original Article	Clinical	21/42	Bilateral VA with collapse of the superior labyrinth (SCs + utricle)	Electrophysiological and MRI findings in patient with BVP.	vHIT: bilateral VOR reduction for all the SCs (ASC spared in four cases, LSC spared in one case). cVEMPs: impaired (5/21). oVEMPa: impaired (19/21).
Marc et al. [6]	2020	Original Article	Clinical	22	Unilateral VA	Description of various clinical presentation of unilateral VA.	Sound-induced vertigo (2/22). BCT: hypo/areflexia. vHIT: VOR reduction for one or more SCs. cVEMPs: abnormal in 54% of cases. oVEMP:s abnormal in 82% of cases.

## Data Availability

The data presented in this study are available on request from the corresponding author. The data are not publicly available due to ethical restrictions.

## Supplementary Materials

The following supporting information can be downloaded at https://www.mdpi.com/article/10.3390/audiolres15030061/s1. Video S1: Video-Frenzel examination of patient n. 1; Video S2: Video-Frenzel examination of patient n. 2.

## Abbreviations

The following abbreviations are used in this manuscript:

AC	Air-conducted
AR	Asymmetry ratio
ASC	Anterior semicircular canal
BCT	Bithermal caloric test
BLB	Blood–labyrinth barrier
BVP	Bilateral vestibulopathy
CSF	Cerebrospinal fluid
cVEMPs	Cervical vestibular-evoked myogenic potentials
EH	Endolymphatic hydrops
FLAIR	Fluid attenuated inversion recovery
HRCT	High-resolution computed tomography
ILS	Intralabyrinthine schwannoma
MD	Meniere’s disease
MRI	Magnetic resonance imaging
LSC	Lateral semicircular canal
oVEMPs	Ocular vestibular-evoked myogenic potentials
PLF	Perilymphatic fistula
PSC	Posterior semicircular canal
SC	Semicircular canal
SNHL	Sensorineural hearing loss
SVINT	Skull vibration-induced nystagmus test
UVL	Unilateral vestibular loss
VA	vestibular atelectasis
VEMPs	Vestibular-evoked myogenic potentials
vHIT	Video head impulse test
VN	Vestibular neuritis
VOR	Vestibulo-ocular reflex
